# Supplementary material for: Association between Immunologic Markers and Cirrhosis in Individuals from a Prospective Chronic Hepatitis C Cohort
Source: Cancers (Basel). 2022 Oct 27;14(21):5280. doi: 10.3390/cancers14215280 (PMC9657502; doi:10.3390/cancers14215280)
Supplement: Supplementary file 1 [file cancers-14-05280-s001.zip › cancers-1935948-supplementary.pdf]

Supplementary Table S1: Odds ratios * and 95% CIs for associations between selected markers and HCC versus cirrhosis.

Analyte	OR (95% CI)			P-value ‡	FDR-corrected P-value ‡
	C2 v. C1	C3 v. C1	C4 v. C1		
HCC vs. Cirrhosis					
**DEFA-1**	0.06 (0.02,0.21)	NA	NA	<0.0001	0.0006
**ITGAM**	0.17 (0.08,0.39)	NA	NA	<0.0001	0.002
**SCF**	1.02 (0.42-2.50)	1.80 (0.63-5.16)	0.72 (0.21-2.47)	0.87	0.95
**CCL11**	3.52 (1.36-9.12)	5.78 (1.97-16.94)	2.50 (0.92-6.79)	0.009	0.15

* Adjusted for age, sex, years of follow-up, serum alanine aminotransferase (ALT) level, alcohol, and smoking
Abbreviations: OR, odds ratio; CI, confidence interval; FDR, false discovery rate; NA, not applicable (marker only has two categories)
‡ N (cirrhosis/HCC): 68/69
‡ P-values calculated as p-trend for analytes with more than two categories

Supplemental Table S2: Odds ratios* and 95%CIs for associations between selected markers and hepatocellular carcinoma (HCC) versus cirrhosis stratified by the presence of underlying cirrhosis in HCC cases

Analyte	Non-cirrhotic HCC cases vs. Cirrhosis ǂ					Non-cirrhotic HCC cases vs. Cirrhosis ǂ			
	**OR (95% CI)**			**P-value‡**		**OR (95% CI)**			**P-value‡**
	**C2 v. Q1**	**C3 v. C1**	**C4 v. C1**			**C2 v. C1**	**C3 v. C1**	**C4 v. C1**	
Cirrhosis v. Control									
DEFA-1	0.08 (0.01-0.62)	NA	NA	0.02		0.06 (0.01-0.21)	NA	NA	<0.0001
ITGAM	0.05 (0.01-0.39)	NA	NA	0.004		0.23 (0.10-0.56)	NA	NA	0.001
SCF	0.97 (0.30-3.18)	0.97 (0.21-4.51)	0.41 (0.04-3.76)	0.49		1.20 (0.42-3.41)	2.39 (0.77-7.46)	0.87 (0.23-3.33)	0.53
CCL11	2.17 (0.61-7.78)	3.69 (0.96-14.21)	0.87 (0.19-4.13)	0.46		4.96 (1.68-14.64)	7.80 (2.31-26.30)	3.71 (1.23-11.20)	0.003

ǂ N (cirrhosis/HCC): 68/25
* Adjusted for age, sex, years of follow-up, serum alanine aminotransferase (ALT) level, alcohol, and smoking
Abbreviations: OR, odds ratio; NA, not applicable (marker only has two categories)

‡ P-values calculated as p-trend for analytes with more than two categories

Supplementary Table S3: Markers tested in REVEAL-HCV; coefficients of variation (CVs) and intraclass correlation coefficients (ICCs) for markers with >90% detection among hepatocellular carcinoma (HCC) cases, cirrhosis cases, or non-cirrhosis controls; and reason for exclusion if excluded from analysis.

Protein	ID	Panel	CV	ICC	Reason if excluded
Adenosine Deaminase (ADA)	P00813	Inflammation	7.43	89.44	
Angiogenin (ANG)	P03950	Cardiometabolic	4.27	83.6	
Angiopoietin-related protein 3 (ANGPTL3)	Q9Y5C1	Cardiometabolic	16.8	63.3	ICC<80%
Apolipoprotein M (APOM)	O95445	Cardiometabolic	14.99	58.57	ICC<80%
Artemin (ARTN)*	Q5T4W7	Inflammation	12.42		
Axin-1 (AXIN1)	O15169	Inflammation	4.21	98.99	
Beta-Ala-His dipeptidase (CNDP1)	Q96KN2	Cardiometabolic	23.86	68.89	ICC<80%
Beta-galactoside alpha-2,6-sialyltransferase 1 (ST6GAL1)	P15907	Cardiometabolic	13.11	57.28	ICC<80%
Beta-nerve growth factor (Beta-NGF)	P01138	Inflammation	2.27	86.64	
Cadherin-1 (CDH1)	P12830	Cardiometabolic	7.73	59.75	ICC<80%
Carbonic anhydrase 1 (CA1)	P00915	Cardiometabolic	5.31	94.4	
Carbonic anhydrase 3 (CA3)	P07451	Cardiometabolic	34.76	98.51	CV>25%
Carbonic anhydrase 4 (CA4)	P22748	Cardiometabolic	33.75	47.39	ICC<80%
Cartilage acidic protein 1 (CRTAC1)	Q9NQ79	Cardiometabolic	19.28	67.4	ICC<80%
Cartilage oligomeric matrix protein (COMP)	P49747	Cardiometabolic	3.9	82.82	
Caspase-8 (CASP-8)	Q14790	Inflammation	2.24	99.33	
C-C motif chemokine 14 (CCL14)	Q16627	Cardiometabolic	4.66	74.5	ICC<80%
C-C motif chemokine 18 (CCL18)	P55774	Cardiometabolic	5.58	90.63	
C-C motif chemokine 19 (CCL19)	Q99731	Inflammation	1.83	97.97	

C-C motif chemokine 20 (CCL20)	P78556	Inflammation	2.58	98.8	
C-C motif chemokine 23 (CCL23)	P55773	Inflammation	2.16	89.35	
C-C motif chemokine 25 (CCL25)	O15444	Inflammation	3.1	96.86	
C-C motif chemokine 28 (CCL28)	Q9NRJ3	Inflammation	5.93	95.82	
C-C motif chemokine 3 (CCL3)	P10147	Inflammation	2.57	99.19	
C-C motif chemokine 4 (CCL4)	P13236	Inflammation	2.34	98.34	
C-C motif chemokine 5 (CCL5)	P13501	Cardiometabolic	10.54	97.23	
CD40L receptor (CD40)	P25942	Inflammation	1.28	97.12	
CD59 glycoprotein (CD59)	P13987	Cardiometabolic	23.68	40.88	ICC<80%
Coagulation factor VII (F7)	P08709	Cardiometabolic	5.9	74.48	ICC<80%
Coagulation factor XI (F11)	P03951	Cardiometabolic	3.67	72.52	ICC<80%
Collagen alpha-1(XVIII) chain (COL18A1)	P39060	Cardiometabolic	10.97	77.51	ICC<80%
Complement C1q tumor necrosis factor-related protein 1 (C1QTNF1)	Q9BXJ1	Cardiometabolic	14.02	68.93	ICC<80%
Complement C2 (C2)	P06681	Cardiometabolic	5.88	83.68	
Complement factor H-related protein 5 (CFHR5)	Q9BXR6	Cardiometabolic	5.16	88.81	
Complement receptor type 2 (CR2)	P20023	Cardiometabolic	4.96	90.57	
CUB domain-containing protein 1 (CDCP1)	Q9H5V8	Inflammation	4.5	97.44	
C-X-C motif chemokine 1 (CXCL1)	P09341	Inflammation	1.42	98.71	
C-X-C motif chemokine 10 (CXCL10)	P02778	Inflammation	2.16	97.99	
C-X-C motif chemokine 11 (CXCL11)	O14625	Inflammation	1.6	95.74	
C-X-C motif chemokine 5 (CXCL5)	P42830	Inflammation	1.58	99.26	
C-X-C motif chemokine 6 (CXCL6)	P80162	Inflammation	2.01	97.27	
C-X-C motif chemokine 9 (CXCL9)	Q07325	Inflammation	2.14	96.28	
Cystatin D (CST5)	P28325	Inflammation	1.74	97.69	
Cystatin-C (CST3)	P01034	Cardiometabolic	4.25	73.52	ICC<80%

Delta and Notch-like epidermal growth factor-related receptor (DNER)	Q8NFT8	Inflammation	1.4	83.45	
Dipeptidyl peptidase 4 (DPP4)	P27487	Cardiometabolic	8.3	66.84	ICC<80%
EGF-containing fibulin-like extracellular matrix protein 1 (EFEMP1)	Q12805	Cardiometabolic	7.15	72.37	ICC<80%
Endoglin (ENG)	P17813	Cardiometabolic	21.02	46.99	ICC<80%
Eotaxin (CCL11)	P51671	Inflammation	0.81	90.05	
Eukaryotic translation initiation factor 4E-binding protein 1 (4E-BP1)	Q13541	Inflammation	4.41	97.57	
Fetuin-B (FETUB)	Q9UGM5	Cardiometabolic	24.77	72.36	ICC<80%
Fibroblast growth factor 19 (FGF-19)	O95750	Inflammation	2.15	98.09	
Fibroblast growth factor 21 (FGF-21)	Q9NSA1	Inflammation	3.76	99.06	
Fibroblast growth factor 23 (FGF-23)	Q9GZV9	Inflammation	5.19	94.46	
Fibroblast growth factor 5 (FGF-5)	Q8NF90	Inflammation			Low detection
Ficolin-2 (FCN2)	Q15485	Cardiometabolic	13.72	79.61	ICC<80%
Fms-related tyrosine kinase 3 ligand (Flt3L)	P49771	Inflammation	1.86	92.17	
Fractalkine (CX3CL1)	P78423	Inflammation	2.73	91.27	
Glial cell line-derived neurotrophic factor (GDNF)	P39905	Inflammation	8.04	73.13	ICC<80%
Glutaminyl-peptide cyclotransferase (QPCT)	Q16769	Cardiometabolic	9.07	69.86	ICC<80%
Granulysin (GNLY)	P22749	Cardiometabolic	18.65	68.82	ICC<80%
Growth arrest-specific protein 6 (GAS6)	Q14393	Cardiometabolic	8.78	66.43	ICC<80%
Hepatocyte growth factor (HGF)	P14210	Inflammation	1.93	90.72	
Hepatocyte growth factor receptor (MET)	P08581	Cardiometabolic	10.83	54.47	ICC<80%
Ig lambda-2 chain C regions (IGLC2)	P0CG05	Cardiometabolic	3.83	79.26	ICC<80%
Insulin-like growth factor-binding protein 3 (IGFBP3)	P17936	Cardiometabolic	5.74	85.43	
Insulin-like growth factor-binding protein 6 (IGFBP6)	P24592	Cardiometabolic	6.79	78.12	ICC<80%
Integrin alpha-M (ITGAM)	P11215	Cardiometabolic	2.55	99.9	

Intercellular adhesion molecule 1 (ICAM1)	P05362	Cardiometabolic	4.13	82.32	
Intercellular adhesion molecule 3 (ICAM3)	P32942	Cardiometabolic	14.65	46.39	ICC<80%
Interferon gamma (IFN-gamma)	P01579	Inflammation			Low detection
Interleukin-1 alpha (IL-1 alpha)	P01583	Inflammation			Low detection
Interleukin-10 (IL10)	P22301	Inflammation	4.94	97.61	
Interleukin-10 receptor subunit alpha (IL-10RA)	Q13651	Inflammation	2.99	99.8	
Interleukin-10 receptor subunit beta (IL-10RB)	Q08334	Inflammation	2.7	73.33	ICC<80%
Interleukin-12 subunit beta (IL-12B)	P29460	Inflammation	2.62	95.93	
Interleukin-13 (IL-13)†	P35225	Inflammation			
Interleukin-15 receptor subunit alpha (IL-15RA)	Q13261	Inflammation	13.8	16.51	ICC<80%
Interleukin-17A (IL-17A)	Q16552	Inflammation	20.09	89.65	
Interleukin-17C (IL-17C)	Q9P0M4	Inflammation	4.19	92.51	
Interleukin-18 (IL-18)	Q14116	Inflammation	2.09	95.53	
Interleukin-18 receptor 1 (IL-18R1)	Q13478	Inflammation	1.89	92.9	
Interleukin-2 (IL-2)‡	P60568	Inflammation			Low detection
Interleukin-2 receptor subunit beta (IL-2RB)	P14784	Inflammation			Low detection
Interleukin-20 (IL-20)	Q9NYY1	Inflammation			Low detection
Interleukin-20 receptor subunit alpha (IL-20RA)	Q9UHF4	Inflammation	5.88	97.79	
Interleukin-22 receptor subunit alpha-1 (IL-22 RA1)	Q8N6P7	Inflammation			Low detection
Interleukin-24 (IL-24)	Q13007	Inflammation	1.39	99.76	
Interleukin-33 (IL-33)	O95760	Inflammation			Low detection
Interleukin-4 (IL-4)	P05112	Inflammation			Low detection
Interleukin-5 (IL5)	P05113	Inflammation	11.27	87.48	
Interleukin-6 (IL6)	P05231	Inflammation	5.94	98.04	
Interleukin-7 (IL-7)	P13232	Inflammation	4.35	99.12	

Interleukin-7 receptor subunit alpha (IL7R)	P16871	Cardiometabolic	19.8	72.54	ICC<80%
Interleukin-8 (IL-8)	P10145	Inflammation	2.72	99.35	
Latency-associated peptide transforming growth factor beta-1 (LAP TGF-beta-1)	P01137	Inflammation	2.11	96.95	
Latent-transforming growth factor beta-binding protein 2 (LTBP2)	Q14767	Cardiometabolic	43.8	23.65	ICC<80%
Leukemia inhibitory factor (LIF)	P15018	Inflammation	8.01	95	
Leukemia inhibitory factor receptor (LIF-R)	P42702	Inflammation	4.53	75.93	ICC<80%
Leukocyte immunoglobulin-like receptor subfamily B member 1 (LILRB1)	Q8NHL6	Cardiometabolic	21.3	81.51	
Leukocyte immunoglobulin-like receptor subfamily B member 2 (LILRB2)	Q8N423	Cardiometabolic	9.42	77.47	ICC<80%
Leukocyte immunoglobulin-like receptor subfamily B member 5 (LILRB5)	O75023	Cardiometabolic	7.68	76.02	ICC<80%
Lithostathine-1-alpha (REG1A)	P05451	Cardiometabolic	3.85	90.18	
Liver carboxylesterase 1 (CES1)	P23141	Cardiometabolic	11.78	92.82	
Low affinity immunoglobulin gamma Fc region receptor II-a (FCGR2A)	P12318	Cardiometabolic	7.01	69.53	ICC<80%
Low affinity immunoglobulin gamma Fc region receptor III-B (FCGR3B)	O75015	Cardiometabolic	5.91	89.83	
L-selectin (SELL)	P14151	Cardiometabolic	4.88	59.52	ICC<80%
Lymphatic vessel endothelial hyaluronic acid receptor 1 (LYVE1)	Q9Y5Y7	Cardiometabolic	5.09	75.23	ICC<80%
Lysosomal Pro-X carboxypeptidase (PRCP)†	P42785	Cardiometabolic			
Macrophage colony-stimulating factor 1 (CSF-1)	P09603	Inflammation	1.38	82.55	
Mannose-binding protein C (MBL2)	P11226	Cardiometabolic	3.2	95.31	
Mast/stem cell growth factor receptor Kit (KIT)	P10721	Cardiometabolic	8.29	74.83	ICC<80%
Matrix metalloproteinase-1 (MMP-1)	P03956	Inflammation	1.3	99.02	
Matrix metalloproteinase-10 (MMP-10)	P09238	Inflammation	2.82	95.27	
Membrane cofactor protein (CD46)	P15529	Cardiometabolic	15.38	61.93	ICC<80%
Membrane primary amine oxidase (AOC3)	Q16853	Cardiometabolic	7.19	78.53	ICC<80%

Metalloproteinase inhibitor 1 (TIMP1)	P01033	Cardiometabolic	4.28	73.59	ICC<80%
Microfibrillar-associated protein 5 (MFAP5))	Q13361	Cardiometabolic	25.28	56.05	ICC<80%
Monocyte chemotactic protein 1 (MCP-1)	P13500	Inflammation	1.86	88.52	
Monocyte chemotactic protein 2 (MCP-2)	P80075	Inflammation	1.98	97.96	
Monocyte chemotactic protein 3 (MCP-3)	P80098	Inflammation	6.04	93.71	
Monocyte chemotactic protein 4 (MCP-4)	Q99616	Inflammation	1.39	92.51	
Multiple epidermal growth factor-like domains protein 9 (MEGF9))	Q9H1U4	Cardiometabolic	17.08	66.88	ICC<80%
Natural killer cell receptor 2B4 (CD244)	Q9BZW8	Inflammation	2.53	91.48	
Neural cell adhesion molecule 1 (NCAM1))	P13591	Cardiometabolic	8.48	67.23	ICC<80%
Neural cell adhesion molecule L1-like protein (CHL1)	O00533	Cardiometabolic	7.88	72.29	ICC<80%
Neurogenic locus notch homolog protein 1 (NOTCH1)	P46531	Cardiometabolic	10.29	59.44	ICC<80%
Neuropilin-1 (NRP1)	O14786	Cardiometabolic	6.08	54.37	ICC<80%
Neurotrophin-3 (NT-3)	P20783	Inflammation	10.54	70.54	ICC<80%
Neurturin (NRTN)	Q99748	Inflammation			Low detection
Neutrophil defensin 1 (DEFA1)	P59665	Cardiometabolic	3.3	99.67	
Neutrophil gelatinase-associated lipocalin (LCN2)	P80188	Cardiometabolic	23.23	44.35	ICC<80%
Nidogen-1 (NID1)	P14543	Cardiometabolic	6.99	76.77	ICC<80%
Oncostatin-M (OSM)	P13725	Inflammation	4.5	99.12	
Oncostatin-M-specific receptor subunit beta (OSMR)	Q99650	Cardiometabolic	6.33	67.29	ICC<80%
Osteoprotegerin (OPG)	O00300	Inflammation	1.68	86.93	
Peptidyl-glycine alpha-amidating monooxygenase (PAM)	P19021	Cardiometabolic	11.13	63.05	ICC<80%
Phospholipid transfer protein (PLTP)	P55058	Cardiometabolic	41.77	50.41	ICC<80%
Plasma serine protease inhibitor (SERPINA5)	P05154	Cardiometabolic	2.81	88.45	
Platelet glycoprotein Ib alpha chain (GP1BA)	P07359	Cardiometabolic	11.62	76.01	ICC<80%

Platelet-activating factor acetylhydrolase (PLA2G7)	Q13093	Cardiometabolic	15.01	62.61	ICC<80%
Plexin-B2 (PLXNB2)	O15031	Cardiometabolic	13.86	60.99	ICC<80%
Procollagen C-endopeptidase enhancer 1 (PCOLCE)	Q15113	Cardiometabolic	4.34	84.24	
Programmed cell death 1 ligand 1 (PD-L1)	Q9NZQ7	Inflammation	3.35	93.95	
Prolyl endopeptidase FAP (FAP)	Q12884	Cardiometabolic	138.24	40.43	ICC<80%
Protein S100-A12 (EN-RAGE)	P80511	Inflammation	6.8	97.68	
Receptor-type tyrosine-protein phosphatase S (PTPRS)	Q13332	Cardiometabolic	17.06	65.65	ICC<80%
Regenerating islet-derived protein 3-alpha (REG3A)	Q06141	Cardiometabolic			Low detection
Serum amyloid A-4 protein (SAA4)	P35542	Cardiometabolic	16.22	71.56	ICC<80%
Signaling lymphocytic activation molecule (SLAMF1)	Q13291	Inflammation	7.54	86.07	
SIR2-like protein 2 (SIRT2)	Q8IXJ6	Inflammation	3.6	99.44	
SPARC-like protein 1 (SPARCL1)	Q14515	Cardiometabolic	14.22	61.17	ICC<80%
STAM-binding protein (STAMPB)	O95630	Inflammation	2.7	99.5	
Stem cell factor (SCF)	P21583	Inflammation	1.1	96.6	
Sulfotransferase 1A1 (ST1A1)	P50225	Inflammation	3.41	99.64	
Superoxide dismutase [Cu-Zn] (SOD1)	P00441	Cardiometabolic	4.53	98.26	
T cell surface glycoprotein CD6 isoform (CD6)	Q8WWJ7	Inflammation	2.78	96.54	
T cell surface glycoprotein CD8 alpha chain (CD8A)	P01732	Inflammation	2.16	95.85	
T-cell immunoglobulin and mucin domain-containing protein 4 (TIMD4)	Q96H15	Cardiometabolic	10.88	67.71	ICC<80%
T-cell surface glycoprotein CD5 (CD5)	P06127	Inflammation	2.45	95.56	
Tenascin (TNC)	P24821	Cardiometabolic	14.5	72.15	ICC<80%
Tenascin-X (TNXB)	P22105	Cardiometabolic	7.32	70.45	ICC<80%
Thrombospondin-4 (THBS4)	P35443	Cardiometabolic	4.89	90.32	
Thymic stromal lymphopoietin (TSLP)	Q969D9	Inflammation			Low detection
Thyroxine-binding globulin (SERPINA7)	P05543	Cardiometabolic	5.42	81.94	

TNF-beta (TNFB)	P01374	Inflammation	2.82	92.65	
TNF-related activation-induced cytokine (TRANCE)	O14788	Inflammation	4.89	94.97	
TNF-related apoptosis-inducing ligand (TRAIL)	P50591	Inflammation	2.27	82.2	
Transcobalamin-2 (TCN2)	P20062	Cardiometabolic	9.44	74.44	ICC<80%
Transforming growth factor alpha (TGF-alpha)	P01135	Inflammation	4.92	94.47	
Transforming growth factor beta receptor type 3 (TGFBR3)	Q03167	Cardiometabolic	20.31	89.85	
Transforming growth factor-beta-induced protein ig-h3 (TGFBI)	Q15582	Cardiometabolic	4.87	66.6	ICC<80%
Trypsin-2 (PRSS2)	P07478	Cardiometabolic	9.35	91.82	
Tumor necrosis factor (Ligand) superfamily, member 12 (TWEAK)	O43508	Inflammation	2.01	90.13	
Tumor necrosis factor (TNF)*	P01375	Inflammation	5.4		
Tumor necrosis factor ligand superfamily member 14 (TNFSF14)	O43557	Inflammation	2.86	98.87	
Tumor necrosis factor receptor superfamily member 9 (TNFRSF9)	Q07011	Inflammation	2.74	87.9	
Tyrosine-protein kinase receptor Tie-1 (TIE1)	P35590	Cardiometabolic	7.83	69.33	ICC<80%
Urokinase-type plasminogen activator (uPA)	P00749	Inflammation	1.81	86.93	
Uromodulin (UMOD)	P07911	Cardiometabolic	0.32	99.85	
Vascular cell adhesion protein 1 (VCAM1)	P19320	Cardiometabolic	6.72	66.96	ICC<80%
Vascular endothelial growth factor A (VEGF-A)	P15692	Inflammation	1.84	95.38	
Vasorin (VASN)	Q6EMK4	Cardiometabolic	11.85	55.92	ICC<80%
Vitamin K-dependent protein C (PROC)	P04070	Cardiometabolic	7.92	64.32	ICC<80%

Supplemental Table S4: Distribution of immunologic markers by pathway

Apoptotic Process	Cell activation involved in	Regulation of immune response	Cell adhesion	Cellular response to cytokine stimulus	Extracellular matrix organization	Inflammatory response	Cellular Metabolic Process	Complement Activation	Immune Response

	**immune response**								
ADA	CCL19	ADA	ADA	CASP_8	COMP	ADA	ANG	ANG	ANG
AXIN1	CCL3	CASP_8	CASP_8	CCL11	ICAM1	CCL11	CA1	CA1	CCL18
BETA_NGF	CD244	CCL19	CCL11	CCL19	ITGAM	CCL19	CCL18	CCL18	CCL5
CASP_8	CD40	CD40	CCL19	CCL20	LAPTGF_B1	CCL20	CCL5	CFHR5	CFHR5
CCL19	CXCL1	CD8A	CCL25	CCL23	MMP_1	CCL23	CES1	CR2	CR2
CCL3	EN_RAGE	IL_12B	CCL28	CCL25	MMP_10	CCL25	ICAM1	ITGAM	DEFA1
CCL5	IL_12B	IL_18R1	CCL4	CCL3	OPG	CCL3	IGFBP3	MBL2	FCGR3B
CD40	IL_18R1	IL10	CCL5	CCL4	PRSS2	CCL4	LILRB1	PRCP	ICAM1
CD5	IL10	IL13	CD5	CD40	TNF	CD40	PRCP	UMOD	ITGAM
COMP	IL13	IL18	CD6	CSF_1		CD6	SOD1		LILRB1
CX3CL1	IL18	IL6	CD8A	CX3CL1		CSF_1	TGFBR3		MBL2
FGF_21	IL6	LAPTGF_B1	COMP	CXCL1		CX3CL1	THBS4		TGFBR3
FLT3L	SLAMF1	PD_L1	CSF_1	CXCL10		CXCL1			
HGF		TNF	CX3CL1	CXCL11		CXCL10			
ICAM1		TNFB	IL_12B	CXCL5		CXCL11			
IGFBP3			IL_18R1	CXCL6		CXCL5			
IL_17A			IL10	CXCL9		CXCL6			
IL_20RA			IL18	FGF_23		CXCL9			
IL_24			IL6	FLT3L		EN_RAGE			
IL10			IL7	HGF		HGF			
IL13			IL8	IL_10RA		IL_12B			
IL6			ITGAM	IL_12B		IL_17A			
IL7			LAPTGF_B1	IL_17A		IL_17C			
LAPTGF_B1			LILRB1	IL_17C		IL_18R1			
LILRB1			MCP_1	IL_18R1		IL10			
MCP_1			PD_L1	IL_20RA		IL13			
OPG			PRSS2	IL_24		IL18			
PD_L1			SCF	IL10		IL5			
SIRT2			SLAMF1	IL13		IL6			
SOD1			SOD1	IL18		IL8			
TNFB			THBS4	IL5		LAPTGF_B1			
TNFRSF9			TNF	IL6		MCP_1			
TNFSF14			TNFSF14	IL8		MCP_2			
TRAIL			TRANCE	LAPTGF_B1		MCP_3			
TWEAK			UMOD	MCP_1		MCP_4			

VEGFA			UPA	MCP_2		OPG			
			VEGFA	MCP_3		OSM			
				MCP_4		TNF			
				MMP_1		TNFB			
				OPG		TNFRSF9			
				OSM		TRANCE			
				TNF					
				TNFB					
				TNFRSF9					
				TNFSF14					
				TRANCE					
				TWEAK					
				VEGFA					

Supplemental Table S5a. P-values and odds ratios (ORs)* and 95% CIs for cirrhotic cases versus controls in REVEAL-HCV for individual markers.

Analyte	Cirrhosis vs control				
	P-value‡	FDR-corrected P-value‡	OR (95% CI)		
			C2 v. C1	C3 v. C1	C4 v. C1
4E_BP1	0.29	0.61	0.54(0.22,1.35)	0.97(0.42,2.25)	0.46(0.17,1.24)
ADA	0.79	0.93	1.11(0.52,2.36)	NA	NA
ANG	0.79	0.93	0.9(0.36,2.23)	0.72(0.28,1.83)	0.94(0.38,2.34)
ARTN	0.01	0.12	21.14(2.4,185.83)	NA	NA
AXIN1	0.25	0.56	0.44(0.16,1.16)	0.57(0.23,1.43)	0.67(0.27,1.63)
BETA_NGF	0.14	0.51	1.83(0.82,4.08)	NA	NA
C2	0.72	0.92	0.67(0.26,1.74)	0.94(0.37,2.35)	1.04(0.43,2.5)
CA1	0.39	0.70	0.4(0.15,1.03)	0.49(0.2,1.23)	0.66(0.28,1.58)
CASP_8	0.04	0.35	0.75(0.31,1.81)	0.61(0.25,1.51)	0.35(0.13,0.95)
CCL11	0.002	0.05	0.3(0.12,0.75)	0.2(0.07,0.57)	0.31(0.12,0.8)
CCL18	0.06	0.39	1.42(0.53,3.8)	1(0.37,2.72)	2.64(1.03,6.73)
CCL19	0.16	0.51	0.51(0.19,1.42)	0.77(0.3,1.97)	1.69(0.72,3.99)
CCL20	0.19	0.51	0.44(0.16,1.23)	1.00(0.4,2.47)	1.54(0.64,3.66)
CCL23	0.21	0.51	0.59(0.24,1.45)	0.68(0.28,1.65)	0.54(0.21,1.37)
CCL25	0.29	0.61	0.88(0.35,2.26)	1.01(0.39,2.58)	1.58(0.65,3.89)
CCL28	0.91	0.98	0.94(0.39,2.23)	0.49(0.18,1.35)	1.13(0.46,2.79)
CCL3	0.07	0.39	1.07(0.41,2.79)	1.28(0.49,3.34)	2.2(0.89,5.44)

CCL4	0.99	1.00	0.59(0.23,1.5)	0.38(0.14,1.05)	1.1(0.47,2.57)
CCL5	0.85	0.95	0.71(0.28,1.79)	0.87(0.35,2.16)	1.04(0.43,2.49)
CD244	0.22	0.51	0.54(0.2,1.45)	0.43(0.15,1.23)	1.63(0.7,3.81)
CD40	0.02	0.20	0.66(0.23,1.9)	1.21(0.46,3.15)	2.65(1.08,6.5)
CD5	0.62	0.88	0.83(0.32,2.12)	0.97(0.39,2.43)	1.21(0.5,2.93)
CD6	0.21	0.51	1.08(0.41,2.83)	1.24(0.48,3.22)	1.73(0.68,4.38)
CD8A	0.41	0.74	1.49(0.63,3.5)	0.65(0.24,1.75)	0.86(0.34,2.16)
CDCP1	0.18	0.51	1.17(0.46,2.97)	1.15(0.44,2.99)	1.91(0.77,4.72)
CES1	0.05	0.35	1.38(0.57,3.31)	2.18(1,4.76)	NA
CFHR5	0.67	0.89	0.71(0.29,1.74)	0.4(0.14,1.09)	0.92(0.39,2.18)
COMP	0.36	0.67	0.68(0.26,1.79)	0.85(0.34,2.12)	1.44(0.6,3.47)
CR2	0.95	0.98	0.51(0.2,1.34)	0.47(0.18,1.24)	0.95(0.4,2.25)
CSF_1	0.33	0.66	1.16(0.47,2.87)	0.52(0.18,1.54)	1.84(0.75,4.53)
CST5	0.92	0.98	1.19(0.5,2.83)	0.67(0.25,1.8)	1.13(0.43,2.94)
CX3CL1	0.72	0.92	0.77(0.31,1.94)	0.58(0.22,1.55)	1.27(0.52,3.14)
CXCL1	0.29	0.61	0.69(0.28,1.71)	0.79(0.33,1.93)	0.58(0.23,1.46)
CXCL10	0.04	0.35	1.5(0.52,4.26)	2.85(1.07,7.58)	2.45(0.94,6.37)
CXCL11	1.0	1.00	0.98(0.39,2.47)	0.6(0.22,1.6)	1.14(0.47,2.75)
CXCL5	0.09	0.41	1.01(0.42,2.41)	0.6(0.24,1.54)	0.49(0.19,1.31)
CXCL6	0.75	0.93	0.93(0.38,2.28)	0.67(0.27,1.71)	0.96(0.4,2.31)
CXCL9	0.22	0.51	1.21(0.46,3.21)	1.65(0.64,4.27)	1.71(0.67,4.36)
DEFA1	<.0001	0.003	7.73(2.96,20.17)	NA	NA
DNER	0.20	0.51	1.08(0.46,2.56)	0.42(0.15,1.14)	0.71(0.29,1.74)
EN_RAGE	0.07	0.39	0.37(0.14,0.95)	0.53(0.22,1.27)	0.41(0.16,1.02)
FCGR3B	0.17	0.51	0.43(0.14,1.33)	1.55(0.64,3.76)	1.38(0.56,3.36)
FGF_19	0.22	0.51	0.79(0.32,1.92)	0.77(0.32,1.84)	0.54(0.21,1.37)
FGF_21	0.35	0.67	0.47(0.19,1.18)	0.49(0.18,1.3)	0.68(0.28,1.67)
FGF_23	0.85	0.95	1.34(0.55,3.26)	1.11(0.44,2.76)	1.09(0.45,2.65)
FLT3L	0.9	0.98	0.86(0.34,2.2)	1.45(0.62,3.39)	0.72(0.27,1.93)
HGF	0.12	0.47	0.64(0.24,1.72)	0.97(0.37,2.52)	1.84(0.76,4.45)
ICAM1	0.09	0.41	0.68(0.24,1.9)	0.98(0.38,2.55)	1.87(0.77,4.53)
IGFBP3	0.47	0.76	1.02(0.41,2.54)	0.57(0.21,1.52)	0.81(0.32,2.07)
IL_10RA	0.44	0.76	1.77(0.8,3.93)	1.19(0.47,3.04)	NA
IL_12B	0.66	0.88	1.54(0.63,3.75)	0.9(0.34,2.38)	1.46(0.56,3.85)

IL_17A	0.87	0.97	0.49(0.19,1.29)	0.88(0.37,2.13)	1.06(0.43,2.58)
IL_17C	0.03	0.35	6.17(1.17,32.51)	NA	NA
IL_18R1	0.18	0.51	1.09(0.41,2.88)	1.06(0.42,2.73)	1.88(0.76,4.66)
IL_20RA	0.49	0.77	1.63(0.4,6.59)	NA	NA
IL_24	0.81	0.94	0.87(0.27,2.82)	NA	NA
IL10	0.34	0.67	1.43(0.58,3.57)	0.92(0.35,2.42)	1.7(0.71,4.06)
IL13	0.47	0.76	1.74(0.39,7.76)	NA	NA
IL18	0.73	0.93	0.94(0.39,2.27)	0.7(0.27,1.78)	0.94(0.38,2.36)
IL5	0.63	0.88	1.39(0.56,3.41)	1.14(0.45,2.89)	NA
IL6	0.25	0.56	1.12(0.44,2.85)	1.49(0.59,3.79)	1.65(0.64,4.26)
IL7	0.79	0.93	0.64(0.25,1.64)	0.69(0.28,1.72)	0.87(0.36,2.1)
IL8	0.05	0.35	1.03(0.39,2.78)	1.9(0.73,4.94)	2.17(0.85,5.55)
ITGAM	0.0002	0.006	4.03(1.94,8.36)	NA	NA
LAPTGF_B1	0.01	0.19	0.67(0.28,1.56)	0.31(0.11,0.83)	0.38(0.14,0.98)
LIF	0.77	0.93	1.18(0.39,3.52)	NA	NA
LILRB1	0.18	0.51	0.75(0.28,2)	0.68(0.26,1.82)	1.72(0.73,4.05)
MBL2	0.45	0.76	1.54(0.6,3.95)	1.16(0.43,3.08)	1.62(0.63,4.14)
MCP_1	0.22	0.51	1.12(0.44,2.85)	0.74(0.27,2.04)	1.9(0.78,4.66)
MCP_2	0.57	0.86	0.69(0.26,1.87)	1.31(0.55,3.13)	1.1(0.44,2.73)
MCP_3	0.63	0.88	0.96(0.37,2.51)	1.48(0.61,3.58)	1.08(0.41,2.82)
MCP_4	0.05	0.35	0.39(0.15,1.01)	0.27(0.1,0.73)	0.54(0.22,1.32)
MMP_1	0.65	0.88	0.33(0.13,0.88)	0.46(0.18,1.2)	0.83(0.36,1.9)
MMP_10	0.51	0.79	0.61(0.25,1.53)	0.56(0.22,1.46)	0.78(0.31,1.96)
OPG	0.10	0.41	1.27(0.47,3.43)	1.25(0.47,3.32)	2.3(0.88,6.01)
OSM	0.95	0.98	0.94(0.38,2.34)	0.74(0.29,1.91)	1.06(0.44,2.52)
PCOLCE	0.18	0.51	0.68(0.28,1.65)	0.37(0.14,0.97)	0.67(0.28,1.62)
PD_L1	0.78	0.93	0.92(0.37,2.29)	0.68(0.26,1.8)	1.26(0.51,3.08)
PRCP	0.01	0.20	7.59(1.5,38.47)	NA	NA
PRSS2	0.99	1.00	1.45(0.59,3.56)	1.2(0.47,3.03)	1.1(0.42,2.89)
REG1A	0.64	0.88	1.34(0.57,3.19)	0.98(0.37,2.56)	0.87(0.34,2.25)
SCF	0.0001	0.006	0.4(0.17,0.94)	0.19(0.07,0.53)	0.19(0.07,0.53)
SERPINA5	0.35	0.67	0.7(0.29,1.69)	0.42(0.16,1.12)	0.76(0.32,1.78)
SERPINA7	0.58	0.87	0.7(0.27,1.86)	1.11(0.45,2.76)	1.11(0.45,2.75)
SIRT2	0.06	0.39	1.24(0.55,2.82)	0.94(0.39,2.26)	0.26(0.08,0.86)

SLAMF1	0.85	0.95	0.74(0.29,1.84)	0.56(0.21,1.46)	1.25(0.51,3.05)
SOD1	0.72	0.92	1.15(0.53,2.51)	NA	NA
ST1A1	0.21	0.51	0.9(0.38,2.16)	0.61(0.23,1.61)	0.61(0.24,1.54)
STAMPB	0.09	0.41	1.03(0.44,2.41)	0.6(0.24,1.52)	0.51(0.2,1.34)
TGF_ALPHA	0.48	0.76	0.43(0.16,1.2)	0.87(0.35,2.15)	1.2(0.52,2.79)
TGFBR3	0.47	0.76	0.82(0.34,2.01)	0.7(0.28,1.72)	0.79(0.32,1.94)
THBS4	0.36	0.67	1.33(0.53,3.34)	0.53(0.18,1.58)	1.79(0.74,4.32)
TNF	0.12	0.47	2.32(0.79,6.78)	NA	NA
TNFB	0.16	0.51	0.88(0.34,2.28)	0.71(0.26,1.93)	1.89(0.78,4.55)
TNFRSF9	0.60	0.88	1.1(0.45,2.73)	0.76(0.29,2.03)	1.44(0.58,3.58)
TNFSF14	0.94	0.98	1.11(0.46,2.72)	0.65(0.24,1.76)	1.17(0.49,2.8)
TRAIL	0.05	0.35	0.43(0.17,1.06)	0.26(0.1,0.7)	0.5(0.21,1.22)
TRANCE	0.12	0.47	1.13(0.49,2.64)	0.73(0.29,1.84)	0.51(0.19,1.33)
TWEAK	0.62	0.88	0.91(0.38,2.18)	0.49(0.19,1.3)	0.96(0.39,2.34)
UMOD	0.47	0.76	1.3(0.51,3.3)	1.28(0.53,3.05)	NA
UPA	0.09	0.41	1.36(0.54,3.44)	0.87(0.31,2.41)	2.49(0.99,6.26)
VEGFA	0.92	0.98	0.54(0.21,1.39)	0.55(0.21,1.42)	1.07(0.46,2.51)

*Adjusted for age, sex, years of follow-up, serum alanine aminotransferase (ALT) level, alcohol, and smoking
‡ P-values calculated as p-trend for analytes with more than two categories
Abbreviations: OR, odds ratio; CI, confidence interval; FDR, false discovery rate; NA, not applicable (marker only has two categories)

Supplemental Table S5b. P-values and odds ratios (ORs)* and 95% CIs for HCC versus cirrhosis cases in REVEAL-HCV for individual markers.

Analyte	HCC vs cirrhosis				
	P-value‡	FDR-corrected P-value‡	OR (95% CI)		
			C2 v. C1	C3 v. C1	C4 v. C1
4E_BP1	0.07	0.44	1.92(0.72,5.17)	1.65(0.68,4.01)	2.92(1.02,8.37)
ADA	0.37	0.76	0.69(0.31,1.55)	NA	NA
ANG	0.77	0.94	1.59(0.61,4.15)	2.14(0.81,5.67)	1.03(0.38,2.79)

ARTN	0.003	0.08	0.09(0.02,0.44)	NA	NA
AXIN1	0.56	0.83	2.18(0.77,6.15)	1.67(0.66,4.26)	1.11(0.43,2.89)
BETA_NGF	0.06	0.37	0.42(0.18,1.02)	NA	NA
C2	0.3	0.76	3.89(1.29,11.72)	4.29(1.49,12.31)	2.09(0.74,5.94)
CA1	0.24	0.76	1.65(0.59,4.59)	1.69(0.65,4.4)	1.67(0.69,4.01)
CASP_8	0.05	0.36	0.53(0.19,1.44)	1.89(0.75,4.74)	2.17(0.76,6.21)
CCL11	0.009	0.15	3.52(1.36,9.12)	5.78(1.97,16.94)	2.5(0.92,6.79)
CCL18	0.002	0.07	0.92(0.33,2.54)	1.11(0.39,3.16)	0.18(0.06,0.51)
CCL19	0.82	0.95	2.34(0.77,7.09)	1.53(0.54,4.32)	1.06(0.42,2.63)
CCL20	0.37	0.76	2.05(0.6,7.03)	2.91(1.08,7.88)	1.52(0.59,3.92)
CCL23	0.34	0.76	1.33(0.52,3.46)	1.83(0.73,4.55)	1.38(0.51,3.79)
CCL25	0.48	0.8	1.02(0.36,2.93)	1.72(0.62,4.76)	1.28(0.5,3.25)
CCL28	0.55	0.83	1.16(0.47,2.88)	2.54(0.89,7.26)	0.54(0.2,1.46)
CCL3	0.73	0.94	1.36(0.47,3.92)	1.39(0.5,3.84)	0.9(0.34,2.38)
CCL4	0.84	0.95	1.26(0.48,3.3)	0.97(0.32,2.93)	0.96(0.41,2.23)
CCL5	0.37	0.76	0.79(0.3,2.08)	0.96(0.37,2.44)	0.61(0.25,1.52)
CD244	0.49	0.81	3.38(1.11,10.26)	3.6(1.14,11.35)	1.78(0.69,4.56)
CD40	0.88	0.95	0.74(0.21,2.6)	1.12(0.41,3.07)	0.86(0.34,2.13)
CD5	0.35	0.76	2.11(0.73,6.08)	2.34(0.83,6.63)	1.76(0.65,4.76)
CD6	0.93	0.97	0.66(0.21,2.03)	1.81(0.67,4.93)	0.82(0.3,2.21)
CD8A	0.29	0.76	1.31(0.53,3.26)	1.54(0.52,4.58)	1.72(0.64,4.59)
CDCP1	0.2	0.65	0.9(0.3,2.71)	1.63(0.56,4.68)	1.6(0.61,4.23)
CES1	0.27	0.76	1.53(0.62,3.81)	1.53(0.7,3.31)	NA
CFHR5	0.02	0.24	0.62(0.25,1.53)	0.69(0.24,2.03)	0.33(0.13,0.84)
COMP	0.39	0.76	1.69(0.57,5.02)	2.58(0.97,6.88)	1.42(0.55,3.71)
CR2	0.46	0.79	4.22(1.47,12.1)	2.88(0.97,8.57)	1.94(0.75,5.03)
CSF_1	0.17	0.64	2.69(0.87,8.28)	7.59(2.17,26.55)	2.39(0.81,7.11)
CST5	0.92	0.97	0.5(0.19,1.3)	1.81(0.66,4.95)	0.7(0.26,1.92)
CX3CL1	0.54	0.83	3.35(1.2,9.34)	3.29(1.09,9.92)	1.69(0.62,4.6)
CXCL1	0.79	0.94	2.01(0.79,5.12)	0.83(0.31,2.2)	1.55(0.57,4.19)
CXCL10	0.46	0.79	0.52(0.15,1.86)	0.7(0.24,2.02)	1.17(0.42,3.25)
CXCL11	0.005	0.11	1.51(0.49,4.66)	5.2(1.69,16.01)	3.35(1.22,9.17)
CXCL5	0.37	0.76	0.88(0.36,2.18)	1.41(0.54,3.7)	1.4(0.5,3.96)
CXCL6	0.42	0.79	1.45(0.57,3.68)	0.96(0.33,2.79)	1.61(0.65,3.98)

CXCL9	0.69	0.94	0.96(0.33,2.77)	0.69(0.24,1.94)	0.87(0.32,2.34)
DEFA1	<.0001	0.0006	0.06(0.02,0.21)	NA	NA
DNER	0.38	0.76	1.33(0.55,3.22)	1.97(0.67,5.82)	1.41(0.54,3.68)
EN_RAGE	0.03	0.24	1.74(0.63,4.8)	2.7(1.1,6.66)	2.39(0.9,6.36)
FCGR3B	0.24	0.76	4.62(1.27,16.84)	1.88(0.66,5.38)	2.63(0.94,7.34)
FGF_19	0.96	0.99	1.17(0.47,2.89)	0.76(0.3,1.93)	1.22(0.45,3.25)
FGF_21	0.14	0.64	2.29(0.88,5.97)	2.34(0.82,6.69)	2(0.78,5.14)
FGF_23	0.47	0.79	0.89(0.34,2.33)	1.75(0.69,4.47)	1.16(0.45,2.99)
FLT3L	0.12	0.6	0.99(0.34,2.9)	1.41(0.57,3.49)	2.24(0.78,6.39)
HGF	0.01	0.2	1.02(0.26,4)	3.03(0.99,9.26)	2.89(1.05,7.99)
ICAM1	0.11	0.59	3.83(1.05,14.03)	3.49(1.04,11.73)	3.27(1.08,9.88)
IGFBP3	0.99	1	1.15(0.45,2.94)	1.79(0.64,4.96)	0.87(0.31,2.42)
IL_10RA	0.87	0.95	1.14(0.52,2.52)	0.9(0.34,2.38)	
IL_12B	0.53	0.83	0.66(0.25,1.74)	1.4(0.5,3.9)	0.57(0.21,1.59)
IL_17A	0.7	0.94	3.04(1.13,8.15)	1.2(0.46,3.13)	1.37(0.53,3.51)
IL_17C	0.75	0.94	0.82(0.25,2.74)	NA	NA
IL_18R1	0.04	0.32	1.91(0.54,6.74)	4.82(1.54,15.1)	2.94(0.99,8.74)
IL_20RA	1	1	1(0.27,3.68)	NA	NA
IL_24	0.45	0.79	0.6(0.16,2.28)	NA	NA
IL10	0.37	0.76	0.78(0.27,2.24)	2.65(0.95,7.45)	1.18(0.46,3.04)
IL13	0.52	0.83	1.55(0.4,6.01)	NA	NA
IL18	0.75	0.94	1.91(0.74,4.9)	1.75(0.62,4.92)	1.28(0.48,3.4)
IL5	0.85	0.95	1.26(0.52,3.05)	1.01(0.38,2.67)	NA
IL6	0.89	0.95	3.8(1.26,11.42)	1.84(0.61,5.54)	1.58(0.51,4.86)
IL7	0.46	0.79	1.31(0.48,3.58)	1.76(0.68,4.58)	1.31(0.52,3.28)
IL8	0.15	0.64	2.49(0.65,9.62)	4.86(1.4,16.93)	2.84(0.81,9.94)
ITGAM	<.0001	0.002	0.17(0.08,0.4)	NA	NA
LAPTGF_B1	0.03	0.24	2.56(1.05,6.25)	3.15(1.09,9.14)	2.93(1.06,8.11)
LIF	0.15	0.64	0.37(0.1,1.43)	NA	NA
LILRB1	0.39	0.76	2.83(0.94,8.5)	2.81(0.93,8.54)	1.95(0.74,5.17)
MBL2	0.39	0.76	0.87(0.33,2.35)	1.11(0.39,3.14)	0.59(0.21,1.63)
MCP_1	0.37	0.76	1.75(0.58,5.25)	4.13(1.33,12.8)	1.6(0.57,4.48)
MCP_2	0.99	1	1.06(0.36,3.16)	1.54(0.63,3.72)	0.84(0.31,2.24)
MCP_3	0.02	0.24	2.57(0.82,8.03)	2.14(0.75,6.1)	4.21(1.41,12.54)

MCP_4	0.03	0.24	6.33(2.3,17.41)	5.34(1.82,15.7)	2.93(1.09,7.88)
MMP_1	0.19	0.64	1.76(0.66,4.74)	0.95(0.34,2.64)	0.57(0.24,1.36)
MMP_10	0.72	0.94	1.88(0.71,4.95)	2.32(0.88,6.12)	0.63(0.22,1.81)
OPG	0.33	0.76	1.44(0.44,4.78)	3.16(1.03,9.7)	1.65(0.55,4.96)
OSM	0.18	0.64	3.12(1.13,8.63)	3.07(1.07,8.79)	2.3(0.86,6.14)
PCOLCE	0.62	0.88	1.38(0.58,3.28)	1.74(0.63,4.82)	0.63(0.23,1.68)
PD_L1	0.08	0.44	2.34(0.79,6.94)	5.11(1.73,15.08)	2.36(0.86,6.49)
PRCP	0.57	0.83	0.73(0.25,2.15)	NA	NA
PRSS2	0.69	0.94	0.73(0.27,1.97)	1.75(0.67,4.59)	0.84(0.29,2.47)
REG1A	0.44	0.79	1.19(0.47,3.01)	1.96(0.71,5.38)	1.28(0.45,3.68)
SCF	0.87	0.95	1.02(0.42,2.5)	1.8(0.63,5.16)	0.72(0.21,2.47)
SERPINA5	0.3	0.76	3.86(1.49,9.99)	3.57(1.2,10.62)	1.96(0.74,5.2)
SERPINA7	0.27	0.76	3.89(1.26,12.04)	2.48(0.85,7.22)	2.59(0.91,7.38)
SIRT2	0.17	0.64	0.8(0.34,1.89)	1.1(0.45,2.72)	3.09(0.86,11.12)
SLAMF1	0.19	0.64	2.09(0.74,5.86)	3.86(1.36,10.98)	1.69(0.65,4.42)
SOD1	0.77	0.94	0.89(0.39,2.01)	NA	NA
ST1A1	0.36	0.76	1.55(0.63,3.85)	1.64(0.58,4.67)	1.57(0.59,4.21)
STAMPB	0.12	0.59	1.9(0.78,4.63)	2.03(0.75,5.49)	2.32(0.82,6.53)
TGF_ALPHA	0.79	0.94	2.55(0.85,7.6)	1.04(0.39,2.76)	1.07(0.45,2.55)
TGFBR3	0.83	0.95	0.82(0.31,2.14)	1.26(0.49,3.22)	0.77(0.3,2.01)
THBS4	0.79	0.94	0.99(0.36,2.71)	1.98(0.59,6.67)	1.04(0.41,2.66)
TNF	0.51	0.82	0.71(0.25,1.99)	NA	NA
TNFB	0.58	0.83	1.13(0.38,3.36)	2.46(0.82,7.33)	1.23(0.47,3.18)
TNFRSF9	0.79	0.94	1.61(0.59,4.37)	1.97(0.67,5.79)	1.19(0.44,3.2)
TNFSF14	0.16	0.64	1.25(0.47,3.33)	2.13(0.72,6.24)	1.8(0.7,4.59)
TRAIL	0.08	0.44	2.03(0.78,5.3)	3.54(1.27,9.87)	1.85(0.73,4.68)
TRANCE	0.82	0.95	1.03(0.44,2.41)	1.02(0.39,2.67)	1.15(0.4,3.29)
TWEAK	0.39	0.76	1.23(0.51,2.95)	1.52(0.55,4.19)	0.56(0.21,1.5)
UMOD	0.73	0.94	2.13(0.87,5.26)	0.88(0.34,2.27)	
UPA	0.96	0.99	1.19(0.42,3.33)	0.99(0.31,3.21)	1.06(0.4,2.78)
VEGFA	0.56	0.83	1.9(0.72,5.05)	1.44(0.53,3.97)	0.79(0.32,1.93)

*Adjusted for age, sex, years of follow-up, serum alanine aminotransferase (ALT) level, alcohol, and smoking
‡ P-values calculated as p-trend for analytes with more than two categories

Abbreviations: OR, odds ratio; CI, confidence interval; FDR, false discovery rate; NA, not applicable (marker only has two categories)
